# Spatial and Temporal Disparities in Timely Hepatitis B Birth-Dose Vaccination in Chongqing, China, 2016–2025

**DOI:** 10.3390/vaccines14080683

**Published:** 2026-08-08

**Authors:** Binyue Xu, Ningyu Wan, Qing Wang, Ningpei Bai, Chunbei Zhou

**Affiliations:** 1 Chongqing Municipal Center for Disease Control and Prevention, Chongqing 400707, China; cqxby@163.com (B.X.);; 2 Chongqing Center of Disease Control and Prevention (Chongqing Academy of Preventive Medicine), Chongqing 400016, China

**Keywords:** hepatitis B vaccination, timely birth-dose vaccination, spatial disparities, GIS, spatiotemporal inequality, western China

## Abstract

Background: Timely hepatitis B vaccine birth-dose (HepBV-BD) vaccination is a cornerstone strategy for preventing mother-to-child transmission of hepatitis B virus (HBV). Although hepatitis B vaccination coverage in China has improved substantially, evidence regarding long-term district-level spatiotemporal inequalities in timely birth-dose vaccination remains limited, particularly in western China. This study assessed spatiotemporal disparities in hepatitis B vaccination coverage across Chongqing, China, from 2016 to 2025. Methods: This ecological descriptive study used district- and county-level vaccination surveillance data from the Chongqing Immunization Information Management System. Temporal trends in HepBV-BD, timely birth-dose (TBD), HepBV2, and HepBV3 coverage were analyzed. Regional disparities, interregional inequalities, and spatial clustering were assessed using Global Moran’s I and Local Indicators of Spatial Association (LISA). Results: All vaccination indicators improved substantially during the study period. TBD coverage increased from 81.9% to 95.4%, whereas HepBV3 coverage increased from 83.7% to 98.7%. TBD coverage was strongly positively associated with HepBV3 coverage(r = 0.95, *p* < 0.001). Mountainous ethnic minority regions consistently exhibited the lowest TBD coverage despite marked improvement over time. Absolute interregional inequality decreased from 30.8 to 8.4 percentage points. Although Global Moran’s I was not statistically significant, LISA analysis identified persistent localized low-coverage clusters in southeastern mountainous regions and emerging high–high clusters in northern Chongqing. Conclusions: Hepatitis B vaccination coverage in Chongqing improved markedly between 2016 and 2025, accompanied by declining geographic inequality. Nevertheless, persistent spatial inequities in timely birth-dose vaccination remained in mountainous ethnic minority areas. Continued monitoring of spatial inequalities, together with strengthened primary healthcare capacity, culturally tailored health education, and equitable resource allocation, may help support geographically targeted immunization strategies and accelerate progress toward the WHO goal of eliminating viral hepatitis as a public health threat by 2030.

## 1. Introduction

The World Health Organization (WHO) has identified viral hepatitis elimination as a major global public health priority [1,2]. Despite remarkable progress in hepatitis B vaccination programs worldwide, hepatitis B virus (HBV) infection remains a leading cause of liver cirrhosis, hepatic failure, and hepatocellular carcinoma, particularly in many low- and middle-income countries in Asia and Africa [3,4]. China has historically borne a heavy burden of HBV infection and remains home to approximately one-third of the global population living with chronic hepatitis B, with an estimated 75 million chronic HBV carriers nationwide [5,6].

Mother-to-child transmission remains one of the principal routes of HBV infection in highly endemic regions [7]. In China, all newborns are required to receive hepatitis B vaccine (HepBV) within 24 h after birth, and infants born to HBsAg-positive mothers must be vaccinated within 12 h to be considered timely vaccination. Timely administration of the HepBV birth dose is recognized as one of the most effective and cost-effective interventions for preventing perinatal HBV transmission [8]. When combined with completion of the subsequent vaccine schedule, timely birth-dose vaccination can substantially reduce the risk of chronic HBV infection in infants and is regarded as a key strategy for achieving the WHO goal of eliminating viral hepatitis as a public health threat by 2030 [1,2]. Although the overall coverage of HepBV among newborns in China has improved substantially over the past decades [9], important gaps remain in timely birth-dose administration and combined immunization among infants born to HBsAg-positive mothers, preterm infants, and children living in remote or underserved regions [10,11]. These gaps represent one of the remaining barriers to achieving hepatitis B elimination in China.

Increasing evidence suggests that substantial regional inequalities persist in HepBV accessibility and timely birth-dose administration across China and other low- and middle-income countries, particularly in rural, western, mountainous, and socioeconomically underdeveloped areas [12,13]. Previous studies conducted in China and other resource-limited settings have demonstrated that disparities in timely birth-dose vaccination are associated with unequal access to maternal and newborn healthcare services, differences in health system capacity, and variations in immunization service delivery [14,15]. These findings highlight the importance of evaluating spatial inequalities in vaccination coverage from a health equity perspective. Identifying geographic areas with persistently low vaccination coverage is essential for supporting equitable allocation of public health resources and monitoring progress toward the WHO immunization Agenda 2030 and the goal of eliminating viral hepatitis as a public health threat [16].

Although previous studies have reported improvements in overall hepatitis B vaccination coverage in China, most analyses have focused on provincial or national averages. Evidence regarding long-term district-level inequalities in timely birth-dose vaccination remains limited, particularly in socioeconomically diverse settings in western China [13]. Moreover, timely birth-dose vaccination may be more sensitive to disparities in perinatal healthcare accessibility than completion of the routine three-dose vaccination schedule. Understanding spatial and temporal inequities in timely birth-dose coverage is therefore essential for identifying vulnerable populations and optimizing targeted immunization strategies.

Chongqing, one of the largest municipalities in western China, encompasses highly urbanized metropolitan districts as well as remote mountainous and ethnic minority counties, providing a unique setting for evaluating geographic disparities in vaccination coverage. To our knowledge, this is the first study to assess decade-long district-level spatiotemporal inequalities in timely HepBV birth-dose vaccination in western China. Therefore, this study aimed to describe temporal trends, regional inequalities, and spatial clustering patterns of hepatitis B vaccination coverage in Chongqing from 2016 to 2025. The findings provide updated epidemiological evidence on district-level spatiotemporal inequalities and may inform equitable allocation of public health resources while guiding future studies into the determinants of spatial disparities.

## 2. Methods

### 2.1. Study Setting and Study Design

This ecological study was conducted in Chongqing, China, from 2016 to 2025, and it was designed to describe long-term temporal trends, regional inequalities, and spatial clustering patterns of HepBV rather than to identify causal determinants of spatial variation. Chongqing is a municipality in southwestern China consisting of 39 districts and counties, including urban districts, peri-urban areas, the Three Gorges Reservoir Area, and mountainous ethnic minority regions. Temporal trends, regional disparities, interregional inequalities, and spatial clustering characteristics of HepBV coverage were analyzed at the district/county level.

Vaccination data were obtained from the Chongqing Immunization Information Management System (CIIMS), the official electronic immunization registry administered by the Chongqing Center for Disease Control and Prevention. The CIIMS routinely collects individual vaccination records reported by all licensed vaccination clinics and delivery institutions throughout Chongqing and has served as the primary surveillance system for the National Immunization Program since 2009.

### 2.2. Data Sources

Data on HepBV coverage were obtained from the CIIMS. Annual district- and county-level vaccination coverage data from 2016 to 2025 were collected for the following indicators: HepBV-BD coverage: hepatitis B vaccine birth-dose coverage; TBD coverage: timely hepatitis B birth-dose vaccination coverage administered within 24 h after birth; HepBV2 coverage: second-dose hepatitis B vaccination coverage; HepBV3 coverage: third-dose hepatitis B vaccination coverage. Before annual reporting, vaccination records undergo routine quality control procedures, including logical consistency checks, duplicate record verification, and completeness assessment conducted by local and municipal CDC staff. The present study used aggregated administrative data without individual-level identifiers. No missing annual vaccination reports were identified across all districts and counties during the 10-year study period. Population statistics and administrative division data were obtained from the Chongqing Statistical Yearbook.

### 2.3. Definitions of Vaccination Indicators

HepBV-BD coverage was defined as the proportion of newborns who received the first dose of HepBV after birth. TBD coverage was defined as the proportion of newborns who received the HepBV within 24 h after birth. HepBV2 and HepBV3 coverage were defined as the proportions of children who completed the second and third doses of HepBV according to the national immunization schedule.

Temporal trend analysis

Annual vaccination coverage rates for HepBV-BD, TBD, HepBV2, and HepBV3 were calculated for each year from 2016 to 2025.

Temporal trends were visualized using line charts. Pearson correlation analysis was conducted to assess the association between TBD coverage and HepBV3 coverage across districts and years. Correlation coefficients (r) and corresponding *p* values were calculated.

Regional comparison analysis: To evaluate regional variation, Chongqing districts and counties were classified into four geographic regions according to official administrative classifications: urban areas; peri-urban areas; the Three Gorges Reservoir Area; mountainous ethnic minority areas. Annual TBD coverage levels were compared among the four regions between 2016 and 2025. Interregional inequality analysis was used to gain insight into absolute and relative interregional inequalities in TBD coverage, assessed annually across all districts and counties. Absolute inequality was measured as the absolute difference between the highest and lowest district-level TBD coverage values each year. Relative inequality was evaluated using the ratio of the highest to the lowest district-level TBD coverage values. In addition, the coefficient of variation (CV) was calculated annually to further quantify relative geographic disparities in vaccination coverage.

Spatial analysis: Spatial distribution patterns of TBD coverage were analyzed at the district/county level using geographic information system (GIS)-based spatial statistical methods. Global spatial autocorrelation was evaluated annually using Global Moran’s I statistics to assess the overall spatial clustering of TBD coverage across Chongqing. Local spatial autocorrelation was assessed using Local Indicators of Spatial Association (LISA) to identify local spatial clusters and spatial outliers, including high–high, low–low, high–low, and low–high clusters. Global Moran’s I was used to evaluate overall spatial autocorrelation across the study area, whereas LISA were applied to identify localized spatial clusters and spatial outliers. Because local clustering may exist even when overall global autocorrelation is weak or statistically nonsignificant, both global and local spatial statistics were performed to provide complementary information regarding the spatial distribution of vaccination coverage. A queen contiguity spatial weight matrix was constructed to define spatial neighborhood relationships among districts and counties. Spatial visualization and spatial statistical analyses were performed using ArcGIS Pro 2.3.0 (Esri, Redlands, CA, USA). Statistical significance for Moran’s I and LISA statistics was assessed using 999 Monte Carlo permutations.

Statistical analysis: Descriptive statistical analyses were performed using SPSS version 25.0 (IBM Corp., Armonk, NY, USA). Vaccination coverage indicators were summarized as percentages. Pearson correlation analysis was used to evaluate the strength of association between timely birth-dose vaccination coverage and HepBV3 coverage across districts and study years. Because of the ecological nature of this study, correlation analyses were interpreted as measures of statistical association and were not intended to infer predictive or causal relationships. Statistical significance was defined as a two-sided *p* < 0.05.

This study was designed as a descriptive ecological analysis. No multivariable regression analyses were performed because the primary objective was to characterize long-term spatiotemporal inequalities in vaccination coverage rather than evaluate determinants of spatial variation.

### 2.4. Ethical Considerations

This study used aggregated administrative data without personal identifiers. Ethical approval was obtained from the Ethics Committee of Chongqing Center for Disease Control and Prevention (KY-2026-039-1, Approval Date: 22 June 2026). All procedures were conducted in accordance with relevant guidelines and regulations.

## 3. Results

### 3.1. Temporal Trends in Hepatitis B Vaccination Coverage

**Figure 1:**
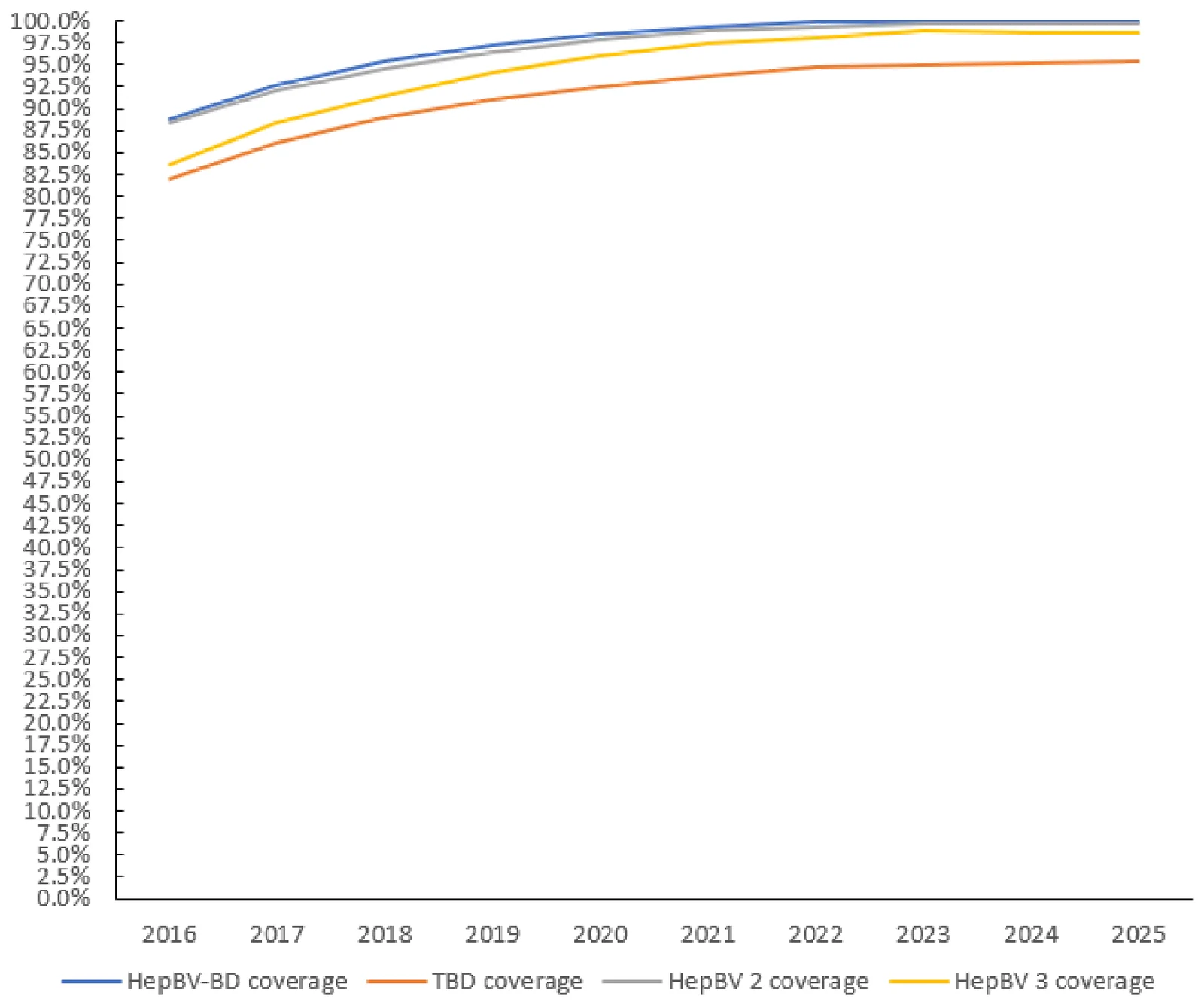
Temporal trends in hepatitis B vaccination coverage in Chongqing, China, 2016–2025.

From 2016 to 2025, hepatitis B vaccination coverage increased across all indicators in Chongqing (Figure 1). HepBV-BD coverage rose from 88.7% to 99.9%, with more rapid increases observed before 2021 and stabilization above 99% after 2023. HepBV2 coverage showed a similar pattern, increasing from 88.3% to 99.7%. HepBV3 coverage increased steadily from 83.7% in 2016 to 98.7% in 2025. TBD coverage also increased continuously, from 81.9% to 95.4% during the study period. Compared with the pre-pandemic period (2016–2019), vaccination coverage remained relatively stable during the COVID-19 pandemic (2020–2022), with only minor year-to-year fluctuations. The rate of improvement slowed slightly during this period but resumed an upward trend after 2022, and no abrupt decline in vaccination coverage was observed during the study period. Across districts and years, timely HepBV birth-dose coverage was strongly positively correlated with HepBV3 coverage (r = 0.945, *p* < 0.001).

### 3.2. Regional Variation in TBD Coverage

**Figure 2:**
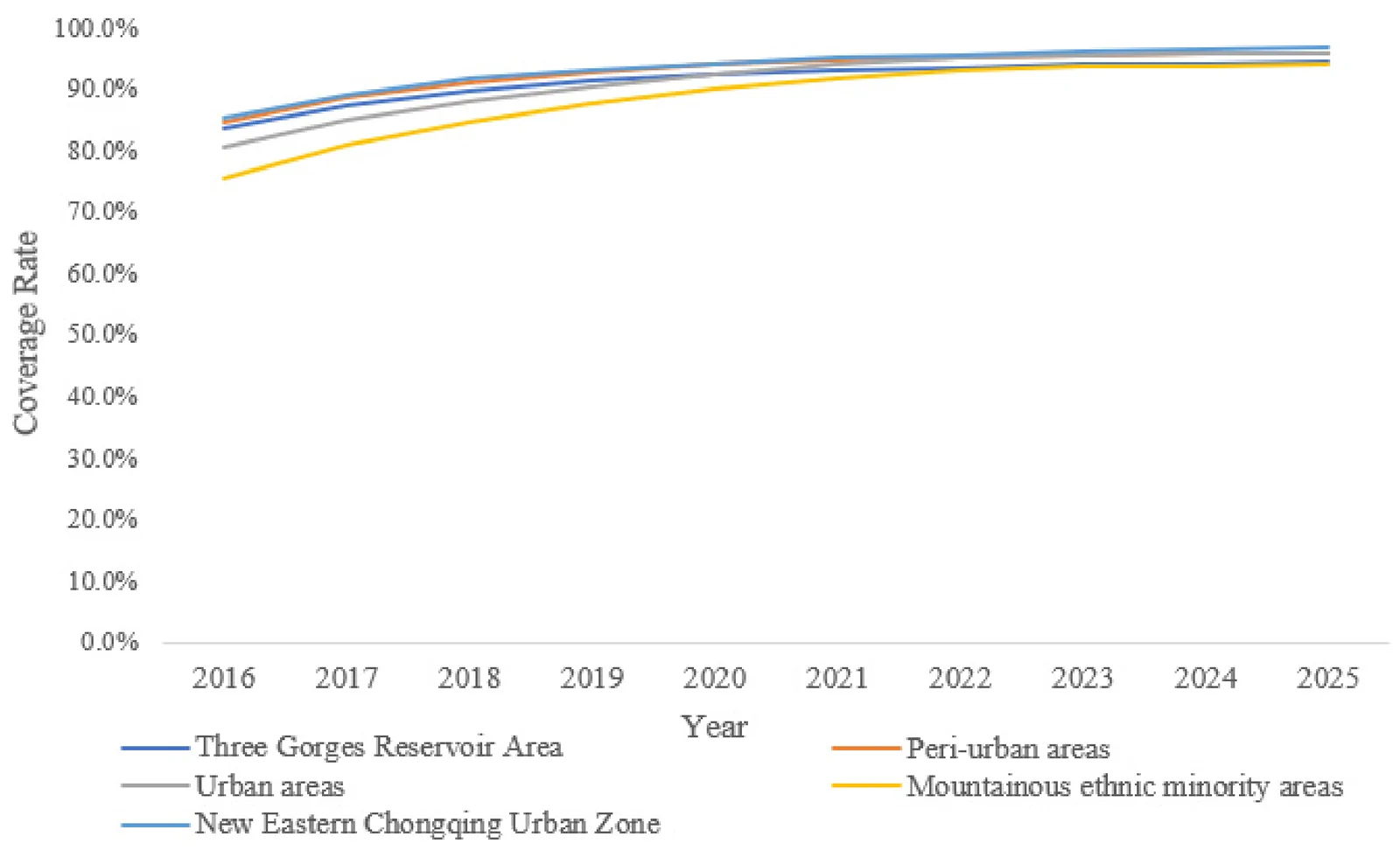
Temporal trends in hepatitis B vaccination coverage across different regions of Chongqing, 2016–2025.

TBD coverage increased across all regions of Chongqing between 2016 and 2025 (Figure 2). Peri-urban areas consistently reported the highest coverage, increasing from 84.6% to 95.9%. Coverage in the Three Gorges Reservoir Area increased from 83.5% to 94.2%, while urban areas increased from 80.3% to 95.7% during the same period. Mountainous ethnic minority areas had the lowest coverage throughout the study period, although coverage increased substantially from 75.6% in 2016 to 94.0% in 2025. Regional disparities narrowed over time; however, lower coverage levels persisted in mountainous ethnic minority regions compared with other areas. Most improvements occurred before 2021, whereas coverage trends became relatively stable thereafter.

### 3.3. Interregional Inequality in TBD Coverage

**Figure 3:**
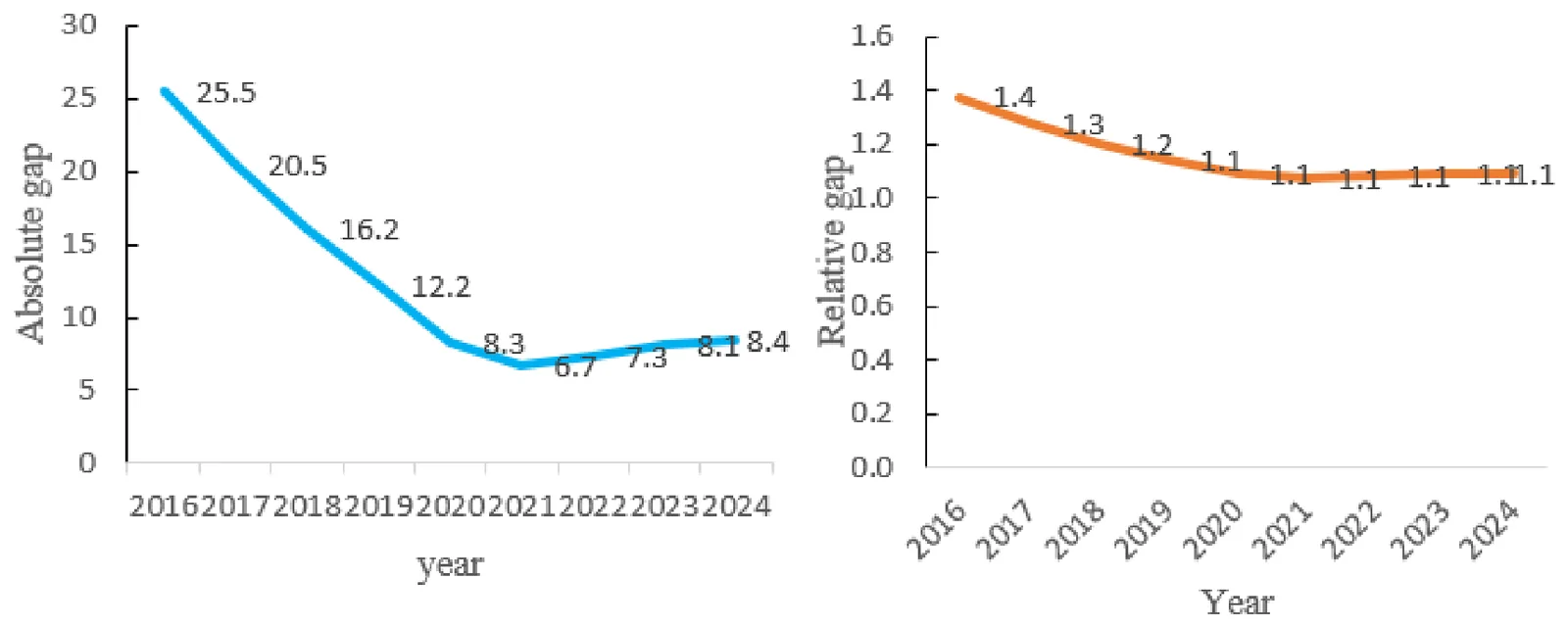
Absolute and relative interregional inequalities in timely HepBV birth-dose vaccination coverage across Chongqing districts and counties, 2016–2025.

Absolute and relative interregional inequalities in TBD coverage decreased continuously between 2016 and 2025 (Figure 3). In 2016, the highest TBD coverage was reported in Kaizhou District (91.2%), whereas the lowest coverage was observed in Youyang Tujia and Miao Autonomous County (60.4%), corresponding to an absolute regional gap of 30.8 percentage points. By 2025, the highest coverage was observed in Nanchuan District (98.1%), while Xiushan Tujia and Miao Autonomous County reported the lowest coverage (89.7%), reducing the absolute gap to 8.4 percentage points. Both absolute and relative measures of inequality showed downward trends throughout the study period, indicating reduced geographic heterogeneity in TBD coverage across districts and counties in Chongqing.

### 3.4. Spatial Clustering Analysis

**Figure 4:**
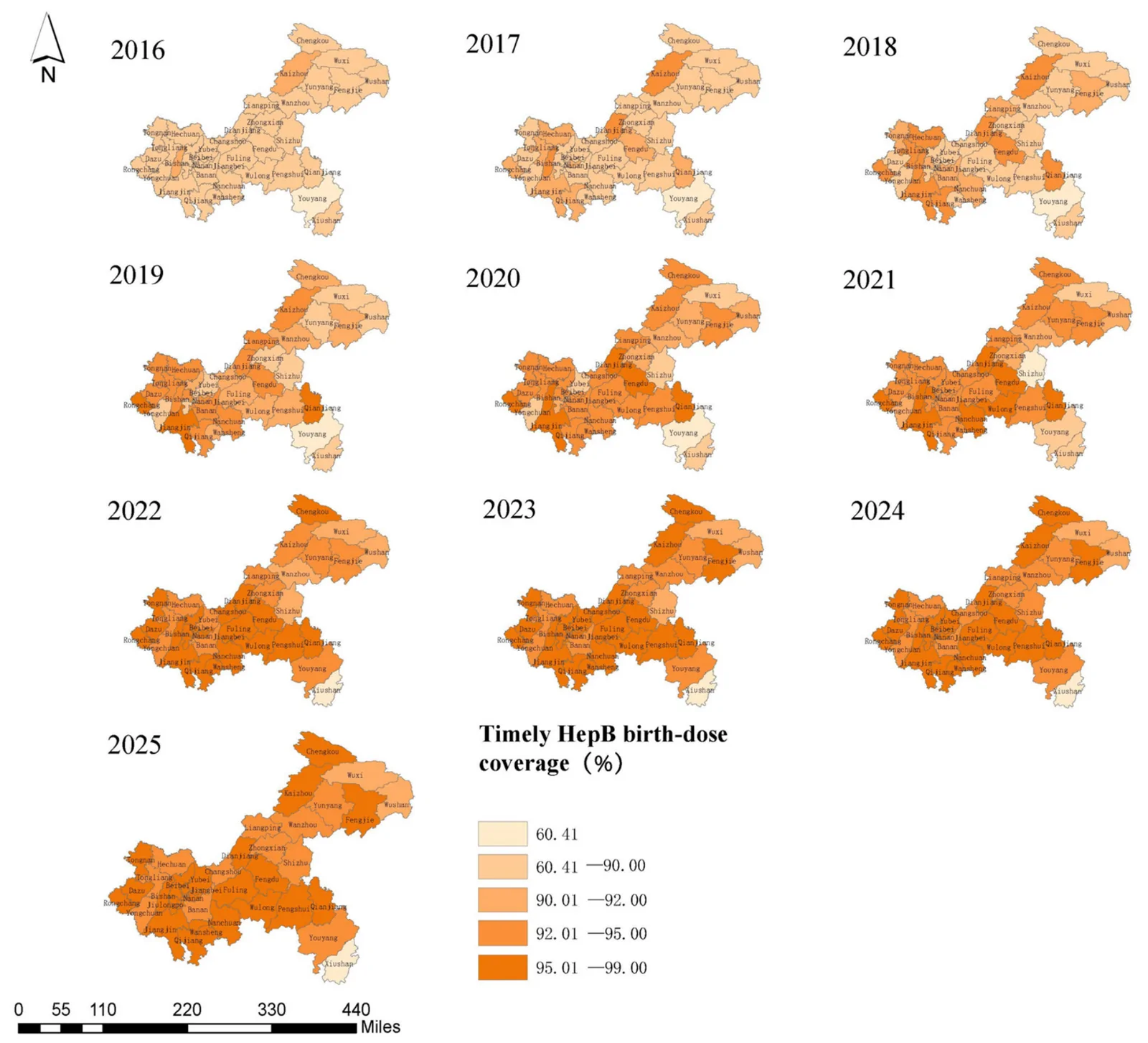
Temporal trends in timely hepatitis B birth-dose vaccination coverage across 39 regions in Chongqing, China, 2016–2025. Reviewed Image ID: GS(2024)0650.

From 2016 to 2025, most districts and counties shifted from moderate TBD coverage levels to high or very high coverage levels (Figure 4). Urban districts generally maintained relatively higher coverage, whereas lower coverage levels were primarily concentrated in ethnic minority and remote mountainous areas in southeastern and northeastern Chongqing. Detailed Local Moran’s I statistics, cluster classifications, and corresponding significance levels are provided in Supplementary Table S1.

**Figure 5:**
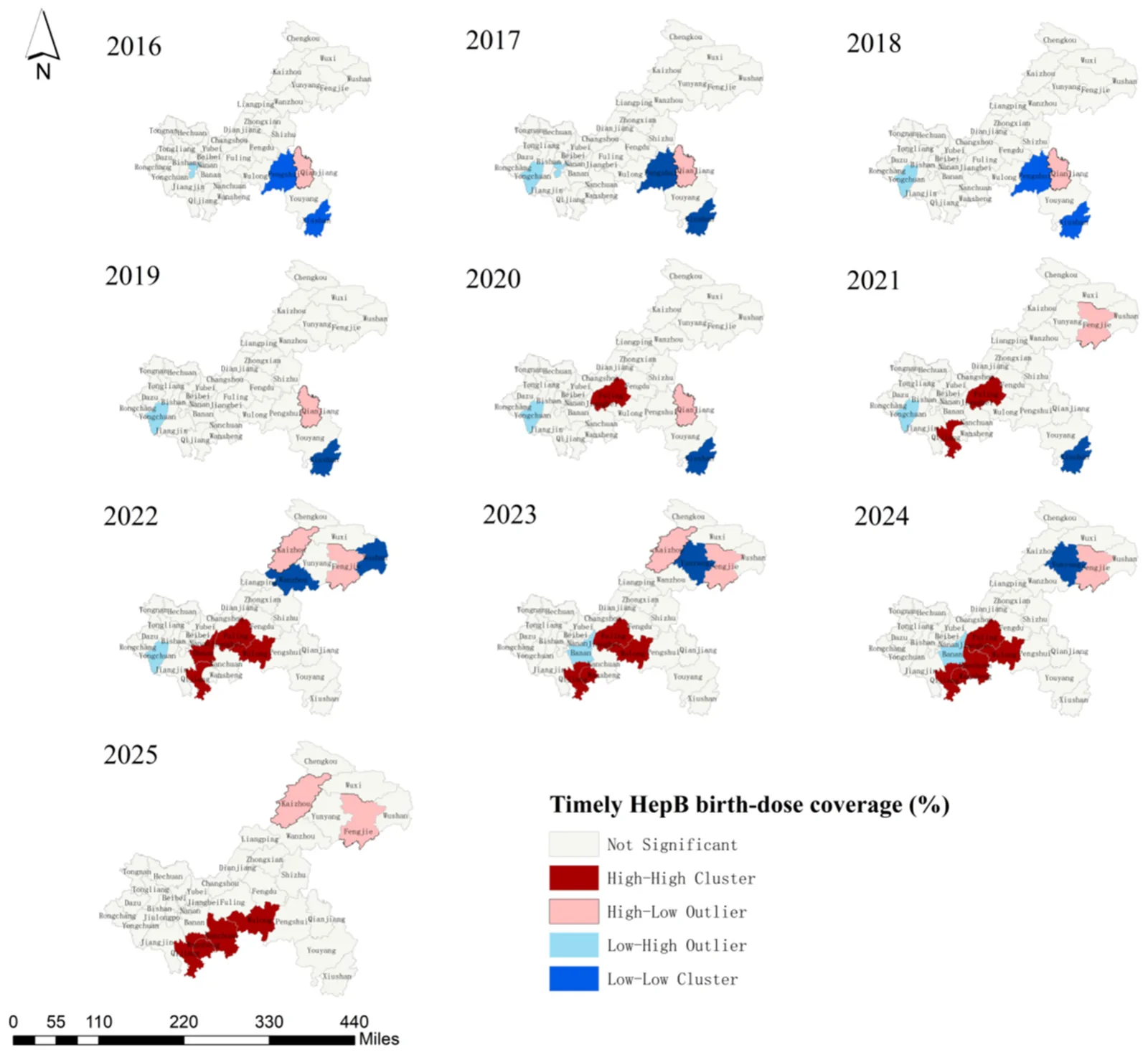
Dynamic Spatial Clustering of Timely Hepatitis B Birth-dose Vaccination Coverage in Chongqing in 2016–2025 Reviewed Image ID: GS(2024)0650.

Global Moran’s I values ranged from −0.03 to 0.08 across study years, and none reached statistical significance (*p* > 0.05), indicating the absence of strong global spatial dependence. However, LISA analysis identified several localized spatial clusters and outlier regions (Figure 5). Early low–low clusters in southeastern Chongqing gradually disappeared over time, whereas high–high clusters became more evident in northern and northeastern Chongqing after 2021. Several low–high and high–low outlier areas were intermittently identified in western and northeastern Chongqing during different study years.

## 4. Discussion

This study presents a decade-long, district-level spatiotemporal inequality analysis of timely neonatal hepatitis B birth-dose vaccination coverage in western China. The findings revealed substantial improvements in vaccination coverage, narrowing geographic inequalities, and persistent vulnerabilities in mountainous ethnic minority regions. Timely birth-dose vaccination coverage was strongly associated with HepBV3 coverage across districts and study years.

Consistent with previous national and international studies [9,17], neonatal hepatitis B vaccination coverage in Chongqing maintained steady growth over the past decade and remained stable despite the global COVID-19 pandemic. The long-term improvement may be associated with the universal neonatal hepatitis B immunization policy, the optimized maternal and child health service system, and strengthened public health promotion and grassroots supervision in western China [9]. Although slight fluctuations were observed during the COVID-19 pandemic, the overall increasing trend was maintained, suggesting that routine hepatitis B immunization services remained largely resilient throughout the pandemic period. Since 2023, the birth-dose vaccination coverage has stabilized above 99%, meeting the immunization targets set by the WHO for hepatitis B elimination [18]. Nevertheless, timely birth-dose vaccination within 24 h still lagged behind other vaccination indicators, indicating that time-limited delivery of the first shot remained a key bottleneck limiting further improvements in neonatal HBV prevention.

Significant regional disparities in timely birth-dose vaccination persisted across Chongqing throughout the study period, with peri-urban districts maintaining the highest coverage and remote mountainous ethnic minority regions reporting the lowest levels. Such spatial heterogeneity aligns with existing evidence of persistent immunization gaps in western inland and ethnic-concentrated areas of China [19,20]. Although the present study did not directly evaluate the determinants of these disparities, previous research suggests that several interconnected factors may contribute to lower vaccination coverage in these remote mountainous areas [14,21]. Geographic remoteness and limited transportation accessibility may delay access to facility-based delivery services [14,21]. Because administering a timely hepatitis B birth dose is particularly challenging when childbirth occurs at home, promotion of facility-based childbirth became a key contributor to China’s success in hepatitis B prevention. Implementation of the timely birth-dose strategy was further accelerated through the GAVI hepatitis B project, which promoted birth-dose vaccination policies in rural and western regions of China [22]. Insufficient grassroots medical workforce and uneven allocation of immunization resources weaken on-site vaccination capacity [23]; meanwhile, traditional health cognition and low awareness of HBV harm among ethnic populations also hinder timely vaccination implementation [24]. The government of China regards health equity as an important component of social justice and fairness, and has continuously promoted equalization of basic public health services in underdeveloped regions [9]. Encouragingly, regional gaps narrowed year by year, possibly reflecting improvements in targeted public health investment and healthcare accessibility.

Interregional absolute and relative inequality metrics presented sustained downward trends, suggesting the gradual convergence of immunization service performance across administrative units in Chongqing. The absolute coverage gap shrank sharply from 30.8 percentage points in 2016 to 8.4 percentage points in 2025, demonstrating substantial progress in narrowing geographic immunization disparity. Spatial analysis revealed no significant global spatial autocorrelation of timely birth-dose vaccination coverage, whereas significant local clustering patterns were observed. Although Global Moran’s I did not indicate significant overall spatial autocorrelation, this does not preclude the presence of localized spatial dependence. Global Moran’s I evaluates the average spatial association across the entire study area, whereas LISA are designed to identify local clusters and spatial outliers. Positive and negative local spatial associations may offset one another when aggregated, resulting in weak or non-significant global autocorrelation despite the existence of meaningful localized clustering. Therefore, the combined use of Global Moran’s I and LISA provides complementary information and enables identification of priority geographic areas for targeted public health interventions. Low–low clustering areas in southeast mountainous regions became less evident, while high–high clusters gradually formed in northern districts, suggesting improving regional clustering of high vaccination coverage [25]. Sporadic spatial outliers indicated that some counties continued to experience localized barriers to timely vaccination despite the performance of neighboring areas. These findings suggest that timely birth-dose vaccination coverage may be a useful population-level measure for monitoring geographic variation in vaccination programme performance.

A strong positive correlation between timely birth-dose vaccination and third-dose completion was observed in this study (r = 0.945, *p* < 0.001), consistent with findings from a national cohort study [26]. However, because this study was based on aggregated ecological data, the observed association should not be interpreted as evidence of causality or predictive ability. Future studies incorporating socioeconomic characteristics, healthcare accessibility, transportation infrastructure, and maternal demographic factors using multivariable analytical approaches are needed to better understand the determinants of spatial disparities in hepatitis B vaccination coverage [27]. Without postexposure prophylaxis provided by the hepatitis B birth dose, approximately 30% of infants born to HBsAg-positive mothers will become infected, and nearly 90% of these infections will progress to chronic hepatitis B infection [28]. Previous epidemiological studies in China have shown that implementation of the nationwide hepatitis B vaccination program was associated with a substantial decline in HBV prevalence among children and adolescents [9,29]. Strengthening timely birth-dose vaccination management should therefore remain a public health priority to improve overall immunization performance and support the prevention of chronic HBV infection among infants.

Given the persistent regional immunization disparities and unique spatial distribution characteristics observed in this study, targeted optimization strategies are proposed to advance hepatitis B elimination in western China. First, priority should be given to supplementing grassroots medical workforce and immunization equipment in mountainous ethnic minority counties, alongside establishing mobile vaccination teams to address geographic accessibility barriers. Second, culturally tailored health education campaigns should be implemented to improve HBV prevention awareness among childbearing-age women and local families in ethnic regions. Third, cross-regional medical cooperation should be strengthened between high-coverage and low-coverage clustering areas to facilitate experience sharing and resource counterpart assistance, thereby eliminating isolated low-immunization zones. Fourth, the 24 h timely birth-dose vaccination rate should be adopted as a core performance assessment indicator to strengthen routine supervision of delivery institutions and minimize missed or delayed neonatal vaccinations. Together, these strategies may help reduce persistent geographic disparities in timely birth-dose vaccination and accelerate progress toward hepatitis B elimination in western China.

### 4.1. Strengths

This study has several strengths. First, it used a comprehensive ten-year longitudinal district-level surveillance dataset covering all districts and counties of Chongqing, enabling fine-scale assessment of hepatitis B vaccination coverage beyond conventional provincial-level analyses. Second, by integrating temporal trend analysis, multidimensional inequality evaluation, and GIS-based spatial clustering approaches, the study comprehensively characterized the spatiotemporal evolution of immunization disparities. Third, Chongqing’s diverse geographic composition, including urban, peri-urban, reservoir, and ethnic mountainous regions, provides valuable public health insights for other socioeconomically heterogeneous areas in western China. Finally, the strong population-level association identified between timely birth-dose vaccination and full-course vaccination completion provides useful epidemiological evidence for optimizing neonatal immunization management.

### 4.2. Limitations

Several limitations should be acknowledged. First, this was an ecological study using aggregated district-level data; therefore, causal inferences at the individual level cannot be made. Second, socioeconomic characteristics, healthcare accessibility, transportation infrastructure, and maternal demographic factors were not available and therefore were not included in the analysis. Third, although strong correlations were observed between timely birth-dose vaccination and HepBV3 coverage, these associations should not be interpreted as predictive relationships. Finally, localized spatial clusters identified by LISA require further investigation using more detailed individual- and community-level data.

## 5. Conclusions

Hepatitis B vaccination coverage in Chongqing improved substantially between 2016 and 2025, accompanied by a continuous reduction in interregional immunization inequality. This long-term district-level spatiotemporal assessment nevertheless identified persistent geographic inequities in timely birth-dose vaccination, with localized low-coverage clusters remaining evident in several mountainous and ethnic minority districts. Timely birth-dose vaccination was strongly associated with completion of the hepatitis B vaccination series at the population level. Continued monitoring of geographic inequalities may help identify priority areas for targeted immunization interventions and inform equitable allocation of public health resources. Future studies incorporating socioeconomic characteristics, healthcare accessibility, and demographic factors are warranted to better understand the determinants of persistent spatial disparities and to support progress toward the WHO goal of eliminating viral hepatitis as a public health threat by 2030.

## Data Availability

The data presented in this study are available on request from the corresponding author.

## Supplementary Materials

The following supporting information can be downloaded at: https://www.mdpi.com/article/10.3390/vaccines14080683/s1, Table S1: District-level Local Moran’s I statistics for TBD vaccination coverage in Chongqing, 2016–2025.
