# Supplementary material for: Spatial and Temporal Disparities in Timely Hepatitis B Birth-Dose Vaccination in Chongqing, China, 2016–2025
Source: Vaccines (Basel). 2026 Aug 8;14(8):683. doi: 10.3390/vaccines14080683 (PMC13517698; doi:10.3390/vaccines14080683)
Supplement: Supplementary file 1 [file vaccines-14-00683-s001.zip › vaccines-4457947-supplementary.pdf]

Supplementary Table S1 District-level Local Moran's I statistics for TBD vaccination coverage in Chongqing, 2016–2025

S1A. District-level Local Moran's I statistics for TBD vaccination coverage in Chongqing, 2016–2019

| district | 2016 TBD(%) | Local Moran's I | Z Score | P value | Cluster type | 2017 TBD(%) | Local Moran's I | Z Score | P value | Cluster type | 2018 TBD(%) | Local Moran's I | Z Score | P value | Cluster type | 2019 TBD(%) | Local Moran's I | Z Score | P value | Cluster type |
|---|---|---|---|---|---|---|---|---|---|---|---|---|---|---|---|---|---|---|---|---|
| Wuxi | 74.67 | -0.114 | -0.225 | 0.444 | | 80.37 | -0.136 | -0.229 | 0.450 | | 84.32 | -0.101 | -0.201 | 0.452 | | 87.19 | -0.051 | -0.054 | 0.480 | |
| Chengkou | 80.12 | -0.046 | -0.229 | 0.414 | | 85.06 | -0.017 | -0.076 | 0.496 | | 88.13 | 0.005 | 0.049 | 0.416 | | 90.95 | 0.013 | 0.281 | 0.316 | |
| Fengjie | 85.55 | -0.464 | -1.455 | 0.084 | | 88.23 | -0.264 | -1.225 | 0.124 | | 90.25 | -0.177 | -1.098 | 0.140 | | 91.79 | -0.120 | -1.268 | 0.126 | |
| Xiushan | 80.53 | 0.718 | 3.674 | 0.024 | LL | 85.1 | 0.777 | 3.512 | 0.030 | LL | 87.56 | 1.363 | 3.749 | 0.026 | LL | 89.01 | 2.449 | 3.879 | 0.022 | LL |
| Pengshui | 76.8 | 0.613 | 1.984 | 0.036 | LL | 82.65 | 0.585 | 2.097 | 0.034 | LL | 86.86 | 0.429 | 1.943 | 0.042 | LL | 90.11 | 0.256 | 1.794 | 0.056 | |
| Dianjiang | 89.57 | 0.411 | 0.895 | 0.186 | | 92.14 | 0.362 | 0.829 | 0.214 | | 93.72 | 0.334 | 0.800 | 0.222 | | 94.9 | 0.263 | 0.649 | 0.288 | |
| Yunyang | 74.57 | -0.400 | -0.731 | 0.246 | | 81.88 | -0.171 | -0.492 | 0.342 | | 86.72 | -0.035 | -0.087 | 0.480 | | 89.86 | 0.047 | 0.259 | 0.364 | |
| Shizhu | 74.06 | -0.215 | -0.316 | 0.392 | | 78.18 | -0.184 | -0.083 | 0.490 | | 81.33 | -0.131 | -0.024 | 0.472 | | 83.61 | 0.041 | 0.223 | 0.368 | |
| Zhongxian | 81.55 | -0.020 | -0.928 | 0.188 | | 85.53 | -0.038 | -0.595 | 0.296 | | 88.03 | -0.047 | -0.373 | 0.382 | | 89.61 | -0.006 | 0.020 | 0.474 | |
| Fengdu | 88.67 | -0.406 | -1.049 | 0.156 | | 91.45 | -0.404 | -1.019 | 0.168 | | 93.48 | -0.370 | -0.840 | 0.206 | | 94.73 | -0.363 | -0.731 | 0.244 | |
| Youyang | 60.41 | -0.036 | 0.138 | 0.434 | | 67.63 | -0.135 | 0.003 | 0.484 | | 73.76 | -0.227 | 0.074 | 0.432 | | 79.67 | -0.225 | 0.100 | 0.446 | |
| Wushan | 81.14 | 0.032 | 0.429 | 0.306 | | 86.09 | 0.008 | 0.487 | 0.270 | | 88.45 | 0.066 | 0.603 | 0.222 | | 89.95 | 0.212 | 0.857 | 0.184 | |
| Tongliang | 88.98 | 0.567 | 1.307 | 0.096 | | 91.12 | 0.419 | 1.137 | 0.118 | | 92.21 | 0.302 | 0.988 | 0.184 | | 93.21 | 0.241 | 0.953 | 0.164 | |
| Tongnan | 85.79 | 0.415 | 1.313 | 0.082 | | 89.52 | 0.394 | 1.127 | 0.116 | | 91.95 | 0.391 | 0.980 | 0.150 | | 93.47 | 0.370 | 1.001 | 0.148 | |
| Hechuan | 88.67 | 0.323 | 0.874 | 0.176 | | 91.17 | 0.298 | 0.781 | 0.228 | | 92.79 | 0.221 | 0.664 | 0.274 | | 93.56 | 0.163 | 0.691 | 0.276 | |
| Changshou | 83.82 | -0.010 | -0.008 | 0.470 | | 86.93 | 0.001 | 0.028 | 0.472 | | 89.26 | 0.001 | 0.025 | 0.454 | | 91.03 | -0.004 | -0.245 | 0.420 | |
| Bishan | 90.28 | 0.648 | 1.577 | 0.058 | | 93.14 | 0.507 | 1.216 | 0.106 | | 94.23 | 0.372 | 0.912 | 0.182 | | 94.88 | 0.320 | 0.887 | 0.188 | |
| Rongchang | 80.21 | 0.063 | 0.305 | 0.342 | | 90.39 | -0.324 | -0.527 | 0.282 | | 94.22 | -0.532 | -0.576 | 0.230 | | 95.83 | -0.410 | -0.436 | 0.260 | |
| Yuzhong | 78.59 | -0.007 | -0.003 | 0.462 | | 82.67 | 0.013 | 0.123 | 0.404 | | 85.88 | 0.019 | 0.067 | 0.430 | | 88.47 | -0.050 | -0.145 | 0.486 | |
| Dazu | 80.76 | -0.051 | -0.578 | 0.278 | | 85.66 | -0.046 | -0.899 | 0.192 | | 89.19 | 0.012 | 0.958 | 0.160 | | 92.09 | 0.126 | 1.012 | 0.132 | |
| Dadukou | 85.4 | 0.272 | 0.898 | 0.188 | | 88.8 | 0.199 | 0.725 | 0.240 | | 90.8 | 0.150 | 0.586 | 0.290 | | 92.37 | 0.122 | 0.653 | 0.272 | |

| | | | | | | | | | | | | | | | | | | | | |
|---|---|---|---|---|---|---|---|---|---|---|---|---|---|---|---|---|---|---|---|---|
| Nanchuan | 83.48 | -0.059 | -0.564 | 0.274 | | 88.21 | -0.062 | -0.388 | 0.304 | | 91.02 | 0.027 | 0.160 | 0.464 | | 92.93 | 0.119 | 0.561 | 0.320 | |
| Jiulongpo | 80.3 | -0.155 | -1.777 | 0.026 | LH | 84.03 | -0.246 | -1.542 | 0.040 | LH | 86.81 | -0.292 | -1.498 | 0.066 | | 89.18 | -0.318 | -1.512 | 0.054 | |
| Yongchuan | 79.93 | -0.193 | -1.413 | 0.074 | | 82.68 | -0.565 | -1.962 | 0.012 | LH | 85.44 | -0.778 | -2.106 | 0.008 | LH | 88.5 | -0.788 | -2.132 | 0.002 | LH |
| Liangping | 84.67 | 0.241 | 0.966 | 0.156 | | 89.17 | 0.251 | 0.794 | 0.244 | | 91.58 | 0.176 | 0.569 | 0.302 | | 92.87 | 0.055 | 0.238 | 0.446 | |
| Fuling | 81.41 | -0.025 | -0.814 | 0.228 | | 85.92 | -0.020 | -0.954 | 0.172 | | 89.18 | 0.011 | 1.217 | 0.116 | | 91.64 | 0.065 | 1.350 | 0.074 | |
| Wanzhou | 84.9 | -0.050 | -0.276 | 0.380 | | 87.57 | -0.036 | -0.274 | 0.354 | | 89.09 | -0.001 | -0.530 | 0.270 | | 90.23 | 0.113 | 0.874 | 0.190 | |
| Qijiang | 86.13 | 0.161 | 0.604 | 0.288 | | 90.54 | 0.208 | 0.583 | 0.316 | | 93.1 | 0.356 | 0.773 | 0.240 | | 94.12 | 0.437 | 1.040 | 0.146 | |
| Kaizhou | 91.19 | -0.766 | -0.956 | 0.162 | | 92.79 | -0.622 | -0.889 | 0.194 | | 93.65 | -0.540 | -0.968 | 0.164 | | 94.25 | -0.482 | -1.020 | 0.166 | |
| Jiangjin | 89.13 | 0.489 | 1.279 | 0.100 | | 91.78 | 0.383 | 1.034 | 0.144 | | 93.78 | 0.322 | 0.766 | 0.242 | | 95.23 | 0.275 | 0.721 | 0.258 | |
| Qianjiang | 88.5 | -2.185 | -2.994 | 0.012 | HL | 91.42 | -2.199 | -3.086 | 0.012 | HL | 93.57 | -2.310 | -2.781 | 0.018 | HL | 95.17 | -2.389 | -2.934 | 0.012 | HL |
| Nanan | 87.8 | -0.276 | -0.589 | 0.240 | | 90.04 | -0.291 | -0.834 | 0.198 | | 91.52 | -0.236 | -0.838 | 0.202 | | 92.7 | -0.160 | -0.700 | 0.222 | |
| Banan | 85.5 | 0.041 | 0.283 | 0.398 | | 88.82 | 0.037 | 0.323 | 0.410 | | 91.09 | 0.038 | 0.338 | 0.378 | | 92.62 | 0.048 | 0.376 | 0.380 | |
| Beibei | 83.7 | 0.030 | 0.293 | 0.384 | | 87.01 | 0.028 | 0.381 | 0.394 | | 89.51 | 0.016 | 0.322 | 0.414 | | 91.64 | 0.020 | 0.296 | 0.418 | |
| Wulong | 73.44 | -0.150 | -0.182 | 0.450 | | 80.66 | -0.181 | -0.329 | 0.398 | | 86.74 | -0.145 | -0.505 | 0.330 | | 90.98 | -0.027 | -0.700 | 0.254 | |
| Shapingba | 84.15 | -0.032 | -0.219 | 0.418 | | 89.02 | -0.087 | -0.397 | 0.316 | | 91.62 | -0.119 | -0.539 | 0.286 | | 93.68 | -0.124 | -0.362 | 0.338 | |
| Jiangbei | 75.61 | 0.147 | 0.385 | 0.304 | | 81.32 | 0.136 | 0.528 | 0.304 | | 85.95 | 0.138 | 0.471 | 0.292 | | 90.1 | 0.061 | 0.508 | 0.266 | |
| Yubei | 70.19 | -0.262 | -0.208 | 0.454 | | 78.08 | -0.114 | -0.074 | 0.478 | | 82.57 | -0.090 | -0.054 | 0.482 | | 86.58 | -0.112 | -0.169 | 0.464 | |
| Wansheng | 75.47 | -0.466 | -0.577 | 0.314 | | 81.03 | -0.625 | -0.883 | 0.178 | | 86.46 | -0.458 | -1.012 | 0.152 | | 90.44 | -0.173 | -1.020 | 0.146 | |
| Liangjiang | 79.37 | 0.216 | 1.245 | 0.102 | | 84.16 | 0.182 | 0.964 | 0.164 | | 87.28 | 0.176 | 0.833 | 0.166 | | 89.92 | 0.091 | 0.447 | 0.296 | |

## S1B. District-level Local Moran's I statistics for TBD vaccination coverage in Chongqing, 2019–2023

| district | 2020 TBD(%) | Local Moran's I | Z Score | P value | Cluster type | 2021 TBD(%) | Local Moran's I | Z Score | P value | Cluster type | 2022 TBD(%) | Local Moran's I | Z Score | P value | Cluster type | 2023 TBD(%) | Local Moran's I | Z Score | P value | Cluster type |
|---|---|---|---|---|---|---|---|---|---|---|---|---|---|---|---|---|---|---|---|---|
| Wuxi | 88.77 | 0.030 | 0.195 | 0.400 | | 89.61 | 0.315 | 0.516 | 0.290 | | 90.27 | 0.714 | 0.939 | 0.184 | | 90.69 | 0.670 | 0.748 | 0.234 | |
| Chengkou | 92.99 | -0.053 | -0.549 | 0.254 | | 94.13 | -0.133 | -1.046 | 0.186 | | 95.31 | -0.444 | -1.532 | 0.082 | | 95.85 | -0.569 | -1.655 | 0.072 | |
| Fengjie | 92.82 | -0.067 | -1.583 | 0.066 | | 93.91 | -0.078 | -1.993 | 0.040 | HL | 94.64 | -0.026 | -2.499 | 0.018 | HL | 95.25 | -0.243 | -2.992 | 0.010 | HL |

| | | | | | | | | | | | | | | | | | | | | |
|---|---|---|---|---|---|---|---|---|---|---|---|---|---|---|---|---|---|---|---|---|
| Xiushan | 89.53 | 3.840 | 3.373 | 0.022 | LL | 89.96 | 4.376 | 2.737 | 0.046 | LL | 90.25 | 3.147 | 1.337 | 0.152 | | 90.08 | 2.297 | 1.073 | 0.174 | |
| Pengshui | 92.32 | 0.067 | 1.347 | 0.102 | | 94.01 | -0.047 | -0.950 | 0.172 | | 95.15 | -0.058 | -0.424 | 0.328 | | 95.91 | -0.055 | -0.225 | 0.406 | |
| Dianjiang | 95.63 | 0.217 | 0.508 | 0.326 | | 96.05 | 0.189 | 0.449 | 0.356 | | 96.39 | 0.159 | 0.518 | 0.326 | | 96.69 | 0.059 | 0.262 | 0.412 | |
| Yunyang | 91.77 | 0.112 | 0.786 | 0.218 | | 93.21 | 0.176 | 1.360 | 0.090 | | 93.96 | 0.336 | 1.910 | 0.054 | | 94.42 | 0.280 | 1.897 | 0.042 | LL |
| Shizhu | 86.12 | 0.301 | 0.452 | 0.294 | | 88.36 | 0.586 | 0.643 | 0.274 | | 90.33 | 0.774 | 0.892 | 0.184 | | 91.46 | 0.647 | 0.785 | 0.232 | |
| Zhongxian | 90.66 | 0.089 | 0.272 | 0.362 | | 91.84 | 0.263 | 0.684 | 0.258 | | 92.84 | 0.403 | 1.011 | 0.156 | | 92.92 | 0.419 | 0.918 | 0.192 | |
| Fengdu | 95.48 | -0.282 | -0.545 | 0.268 | | 95.95 | -0.203 | -0.461 | 0.332 | | 96.12 | -0.087 | -0.192 | 0.396 | | 96.37 | -0.021 | 0.001 | 0.472 | |
| Youyang | 84.17 | 0.010 | 0.231 | 0.382 | | 88.83 | 0.365 | 0.326 | 0.374 | | 92.28 | 0.461 | 0.794 | 0.22 | | 93.51 | 0.407 | 0.975 | 0.178 | |
| Wushan | 90.7 | 0.514 | 1.003 | 0.140 | | 91.24 | 1.183 | 1.440 | 0.110 | | 91.62 | 1.996 | 1.889 | 0.042 | LL | 91.67 | 2.124 | 1.786 | 0.064 | |
| Tongliang | 93.69 | 0.135 | 0.769 | 0.236 | | 93.95 | 0.015 | 0.433 | 0.364 | | 93.97 | -0.005 | -0.042 | 0.494 | | 93.89 | -0.021 | -0.013 | 0.480 | |
| Tongnan | 94.18 | 0.284 | 0.863 | 0.214 | | 94.61 | 0.110 | 0.532 | 0.360 | | 95.01 | -0.002 | -0.106 | 0.422 | | 95.41 | -0.029 | -0.255 | 0.388 | |
| Hechuan | 94.05 | 0.120 | 0.490 | 0.340 | | 94.41 | 0.077 | 0.604 | 0.302 | | 94.56 | -0.008 | -0.685 | 0.284 | | 94.75 | -0.033 | -0.658 | 0.290 | |
| Changshou | 92.43 | -0.010 | -0.247 | 0.436 | | 93.86 | 0.012 | 0.652 | 0.282 | | 95.22 | 0.151 | 0.945 | 0.198 | | 95.06 | 0.023 | 0.941 | 0.184 | |
| Bishan | 95.41 | 0.265 | 0.828 | 0.220 | | 95.84 | 0.222 | 0.741 | 0.258 | | 96.02 | 0.146 | 0.576 | 0.296 | | 96.24 | 0.105 | 0.501 | 0.334 | |
| Rongchang | 96.41 | -0.433 | -0.393 | 0.296 | | 96.67 | -0.577 | -0.548 | 0.288 | | 96.92 | -0.583 | -0.615 | 0.28 | | 97.2 | -0.427 | -0.459 | 0.306 | |
| Yuzhong | 90.53 | -0.128 | -0.275 | 0.448 | | 92.5 | -0.199 | -0.613 | 0.316 | | 94.02 | -0.162 | -1.012 | 0.162 | | 94.41 | -0.156 | -1.111 | 0.144 | |
| Dazu | 93.8 | 0.162 | 0.757 | 0.254 | | 94.64 | 0.067 | 0.308 | 0.414 | | 95.26 | -0.010 | -0.050 | 0.45 | | 95.65 | -0.015 | -0.120 | 0.418 | |
| Dadukou | 93.2 | 0.082 | 0.680 | 0.266 | | 94.27 | 0.103 | 0.792 | 0.242 | | 94.84 | 0.062 | 0.841 | 0.238 | | 95.12 | 0.035 | 0.756 | 0.258 | |
| Nanchuan | 94.49 | 0.263 | 0.953 | 0.184 | | 95.99 | 0.518 | 1.250 | 0.114 | | 96.88 | 0.670 | 1.305 | 0.08 | | 97.4 | 0.765 | 1.496 | 0.058 | |
| Jiulongpo | 91.12 | -0.278 | -1.358 | 0.066 | | 93.3 | -0.110 | -1.394 | 0.066 | | 95.19 | 0.133 | 1.334 | 0.096 | | 95.55 | 0.115 | 1.043 | 0.152 | |
| Yongchuan | 89.88 | -0.993 | -2.149 | 0.006 | LH | 91.19 | -1.035 | -1.891 | 0.016 | LH | 92.33 | -0.805 | -1.450 | 0.05 | LH | 93.1 | -0.581 | -1.234 | 0.114 | |
| Liangping | 93.56 | -0.038 | -0.159 | 0.402 | | 94.15 | -0.059 | -0.560 | 0.270 | | 94.54 | 0.019 | 0.855 | 0.208 | | 94.77 | 0.063 | 0.886 | 0.204 | |
| Fuling | 93.38 | 0.177 | 1.741 | 0.036 | HH | 94.84 | 0.358 | 1.783 | 0.032 | HH | 95.76 | 0.458 | 1.886 | 0.022 | HH | 96.27 | 0.474 | 1.852 | 0.016 | HH |
| Wanzhou | 90.74 | 0.356 | 1.155 | 0.130 | | 91.42 | 0.716 | 1.731 | 0.064 | | 91.99 | 1.058 | 1.827 | 0.05 | LL | 92.39 | 1.005 | 1.772 | 0.052 | |
| Qijiang | 94.49 | 0.450 | 1.384 | 0.074 | | 94.97 | 0.434 | 1.616 | 0.024 | HH | 95.24 | 0.281 | 1.796 | 0.02 | HH | 95.64 | 0.310 | 1.811 | 0.004 | HH |

| | | | | | | | | | | | | | | | | | | | | |
|---|---|---|---|---|---|---|---|---|---|---|---|---|---|---|---|---|---|---|---|---|
| Kaizhou | 94.53 | -0.437 | -1.264 | 0.116 | | 94.74 | -0.378 | -1.500 | 0.074 | | 94.86 | -0.134 | -1.986 | 0.042 | HL | 95.23 | -0.136 | -1.910 | 0.036 | HL |
| Jiangjin | 96.08 | 0.164 | 0.432 | 0.378 | | 96.38 | 0.126 | 0.308 | 0.430 | | 96.57 | 0.066 | 0.218 | 0.414 | | 96.7 | 0.060 | 0.271 | 0.426 | |
| Qianjiang | 95.97 | -2.161 | -2.458 | 0.020 | HL | 96.39 | -1.431 | -1.706 | 0.054 | | 96.51 | -0.527 | -0.660 | 0.254 | | 96.32 | -0.113 | -0.174 | 0.384 | |
| Nanan | 93.62 | -0.100 | -0.405 | 0.334 | | 94.58 | -0.032 | -0.107 | 0.446 | | 95.22 | 0.046 | 0.354 | 0.402 | | 95.51 | 0.038 | 0.222 | 0.476 | |
| Banan | 93.51 | 0.062 | 0.677 | 0.252 | | 94.3 | 0.086 | 1.195 | 0.118 | | 94.68 | 0.020 | 1.928 | 0.02 | HH | 94.82 | -0.043 | -1.786 | 0.026 | LH |
| Beibei | 93.2 | 0.047 | 0.553 | 0.320 | | 94.81 | 0.157 | 0.739 | 0.244 | | 95.91 | 0.290 | 1.036 | 0.172 | | 96.3 | 0.293 | 1.066 | 0.160 | |
| Wulong | 93.57 | 0.183 | 0.957 | 0.182 | | 95.12 | 0.447 | 1.435 | 0.076 | | 96.07 | 0.620 | 1.662 | 0.026 | HH | 96.29 | 0.643 | 1.885 | 0.002 | HH |
| Shapingba | 94.79 | -0.039 | -0.033 | 0.460 | | 95.59 | 0.152 | 0.571 | 0.308 | | 96.07 | 0.317 | 1.070 | 0.15 | | 96.31 | 0.305 | 1.138 | 0.126 | |
| Jiangbei | 92.59 | 0.001 | 0.251 | 0.376 | | 94.31 | 0.013 | 0.150 | 0.476 | | 95.51 | 0.108 | 0.526 | 0.334 | | 95.97 | 0.118 | 0.644 | 0.288 | |
| Yubei | 89.42 | -0.172 | -0.310 | 0.408 | | 92.25 | -0.182 | -0.639 | 0.286 | | 94.79 | 0.032 | 0.823 | 0.222 | | 95.37 | 0.057 | 0.736 | 0.244 | |
| Wansheng | 92.89 | 0.076 | 1.042 | 0.150 | | 94.65 | 0.349 | 1.224 | 0.102 | | 96.05 | 0.648 | 1.267 | 0.056 | | 96.73 | 0.876 | 1.366 | 0.048 | HH |
| Liangjiang | 92.1 | 0.009 | 0.097 | 0.422 | | 94.02 | 0.027 | 0.415 | 0.384 | | 95.2 | 0.176 | 1.119 | 0.132 | | 95.61 | 0.209 | 1.198 | 0.126 | |

## S1C. District-level Local Moran's I statistics for TBD vaccination coverage in Chongqing, 2024–2025

| district | 2024 TBD(%) | Local Moran's I | Z Score | P value | Cluster type | 2025 TBD(%) | Local Moran's I | Z Score | P value | Cluster type |
|---|---|---|---|---|---|---|---|---|---|---|
| Wuxi | 90.8 | 0.598 | 0.715 | 0.222 | | 90.82 | 0.616 | 0.833 | 0.196 | |
| Chengkou | 96.33 | -0.699 | -1.614 | 0.076 | | 96.74 | -0.849 | -1.606 | 0.084 | |
| Fengjie | 95.53 | -0.271 | -2.796 | 0.010 | HL | 95.88 | -0.450 | -2.801 | 0.006 | HL |
| Xiushan | 89.7 | 2.570 | 1.077 | 0.148 | | 89.68 | 3.147 | 1.266 | 0.108 | |
| Pengshui | 96.56 | -0.053 | -0.180 | 0.418 | | 96.89 | -0.027 | -0.058 | 0.448 | |
| Dianjiang | 96.9 | 0.057 | 0.212 | 0.452 | | 97.14 | 0.050 | 0.213 | 0.458 | |
| Yunyang | 94.82 | 0.191 | 1.892 | 0.040 | LL | 94.75 | 0.294 | 1.787 | 0.056 | |
| Shizhu | 92.45 | 0.386 | 0.630 | 0.250 | | 93.77 | 0.142 | 0.344 | 0.330 | |
| Zhongxian | 93.27 | 0.326 | 0.795 | 0.206 | | 93.95 | 0.151 | 0.545 | 0.278 | |
| Fengdu | 96.64 | 0.095 | 0.411 | 0.376 | | 96.76 | 0.223 | 0.882 | 0.208 | |
| Youyang | 93.67 | 0.466 | 0.945 | 0.178 | | 93.5 | 0.588 | 1.070 | 0.160 | |

| | | | | | | | | | | |
|---|---|---|---|---|---|---|---|---|---|---|
| Wushan | 91.63 | 2.217 | 1.750 | 0.068 | | 91.5 | 2.312 | 1.828 | 0.080 | |
| Tongliang | 93.95 | -0.050 | -0.127 | 0.478 | | 94.14 | -0.083 | -0.284 | 0.440 | |
| Tongnan | 95.8 | -0.055 | -0.320 | 0.348 | | 95.93 | -0.040 | -0.262 | 0.338 | |
| Hechuan | 94.82 | -0.054 | -0.582 | 0.304 | | 94.99 | -0.051 | -0.548 | 0.326 | |
| Changshou | 94.81 | -0.104 | -0.947 | 0.174 | | 94.34 | -0.265 | -0.974 | 0.172 | |
| Bishan | 96.33 | 0.074 | 0.388 | 0.382 | | 96.48 | 0.069 | 0.291 | 0.414 | |
| Rongchang | 97.45 | -0.227 | -0.234 | 0.366 | | 97.73 | -0.048 | -0.077 | 0.392 | |
| Yuzhong | 94.75 | -0.106 | -0.905 | 0.208 | | 94.81 | -0.103 | -0.679 | 0.278 | |
| Dazu | 95.93 | 0.010 | 0.108 | 0.472 | | 96.26 | 0.043 | 0.143 | 0.498 | |
| Dadukou | 95.41 | 0.029 | 0.462 | 0.384 | | 95.51 | 0.010 | 0.322 | 0.424 | |
| Nanchuan | 97.78 | 0.826 | 1.591 | 0.044 | HH | 98.07 | 0.863 | 1.554 | 0.042 | HH |
| Jiulongpo | 95.71 | 0.073 | 0.802 | 0.220 | | 95.63 | 0.027 | 0.586 | 0.296 | |
| Yongchuan | 93.82 | -0.357 | -1.072 | 0.180 | | 94.35 | -0.261 | -1.076 | 0.142 | |
| Liangping | 94.76 | 0.133 | 1.092 | 0.138 | | 94.7 | 0.155 | 0.888 | 0.208 | |
| Fuling | 97.12 | 0.564 | 1.598 | 0.044 | HH | 97.6 | 0.539 | 1.325 | 0.078 | |
| Wanzhou | 92.52 | 0.855 | 1.399 | 0.096 | | 92.7 | 0.630 | 1.282 | 0.108 | |
| Qijiang | 96.02 | 0.338 | 1.635 | 0.022 | HH | 96.33 | 0.377 | 1.528 | 0.036 | HH |
| Kaizhou | 95.5 | -0.140 | -1.699 | 0.064 | | 95.71 | -0.160 | -2.018 | 0.040 | HL |
| Jiangjin | 96.63 | 0.053 | 0.150 | 0.472 | | 96.59 | 0.044 | 0.282 | 0.434 | |
| Qianjiang | 96.37 | -0.033 | 0.013 | 0.420 | | 96.36 | -0.052 | -0.124 | 0.390 | |
| Nanan | 95.65 | 0.016 | 0.213 | 0.460 | | 95.71 | -0.003 | 0.027 | 0.466 | |
| Banan | 94.7 | -0.135 | -1.849 | 0.026 | LH | 94.69 | -0.158 | -1.444 | 0.076 | |
| Beibei | 96.7 | 0.269 | 0.855 | 0.214 | | 97.02 | 0.253 | 0.805 | 0.240 | |
| Wulong | 96.27 | 0.583 | 2.087 | 0.006 | HH | 96.18 | 0.464 | 2.171 | 0.004 | HH |
| Shapingba | 96.37 | 0.255 | 1.048 | 0.146 | | 96.38 | 0.200 | 0.913 | 0.192 | |

| | | | | | | | | | | |
|---|---|---|---|---|---|---|---|---|---|---|
| Jiangbei | 96.16 | 0.083 | 0.431 | 0.386 | | 96.26 | 0.042 | 0.256 | 0.420 | |
| Yubei | 95.55 | 0.033 | 0.408 | 0.382 | | 95.54 | 0.009 | 0.241 | 0.454 | |
| Wansheng | 97.24 | 1.040 | 1.389 | 0.034 | HH | 97.5 | 1.144 | 1.450 | 0.022 | HH |
| Liangjiang | 95.96 | 0.224 | 1.190 | 0.122 | | 96.09 | 0.195 | 1.022 | 0.150 | |
